# Preoperative assessment and evaluation of instrumentation strategies for the treatment of adolescent idiopathic scoliosis: computer simulation and optimization

**DOI:** 10.1186/1748-7161-7-21

**Published:** 2012-11-26

**Authors:** Younes Majdouline, Carl-Eric Aubin, Xiaoyu Wang, Archana Sangole, Hubert Labelle

**Affiliations:** 1 Department of Mechanical Engineering, École Polytechnique, Universite de Montreal, P.O. Box 6079, Downtown Station, Montréal, Québec H3C 3A7, Canada; 2 Research Center, Sainte-Justine University Hospital Center of Universite de Montreal, 3175, Cote Sainte-Catherine Rd, Montréal, Québec H3T 1C5, Canada

**Keywords:** Scoliosis, Instrumentation, Simulation, Modeling, Optimization, 3-D correction

## Abstract

**Background:**

A large variability in adolescent idiopathic scoliosis (AIS) correction objectives and instrumentation strategies was documented. The hypothesis was that different correction objectives will lead to different instrumentation strategies. The objective of this study was to develop a numerical model to optimize the instrumentation configurations under given correction objectives.

**Methods:**

Eleven surgeons from the Spinal Deformity Study Group independently provided their respective correction objectives for the same patient. For each surgeon, 702 surgical configurations were simulated to search for the most favourable one for his particular objectives. The influence of correction objectives on the resulting surgical strategies was then evaluated.

**Results:**

Fusion levels (mean 11.2, SD 2.1), rod shapes, and implant patterns were significantly influenced by correction objectives (p < 0.05). Different surgeon-specified correction objectives produced different instrumentation strategies for the same patient.

**Conclusions:**

Instrumentation configurations can be optimized with respect to a given set of correction objectives.

## Background

Adolescent idiopathic scoliosis (AIS) is a three-dimensional (3D) local and global deformation of the spine [1], which may require spinal instrumentation and fusion for severe cases [2]. The main objectives of the surgical procedure are to correct the deformity, to obtain a balanced posture and preserve spinal mobility [3]. The strategies to achieve these objectives are based on an accurate selection of fusion levels and an adequate application of corrective forces through spinal instrumentation [4,5].

In recent years, many changes have occurred for the surgical treatment of scoliosis. With contemporary advanced instrumentation systems and techniques, surgeons have a wide range of choices to achieve the goals of surgery, such as various implant types, diverse rod materials, diameter and shape possibilities as well as many intraoperative reduction manoeuvres. The surgical decision-making process has considerably increased in complexity, with many on-going controversies and debates over the choices of fusion levels, the proper guidelines for surgical correction and the choice of the instrumentation system [6-8]. Three previous studies have documented a large variability in AIS instrumentation strategies, and in the correction objectives in a group of experienced spine surgeons [1,9,10]. Different instrumentation strategies and selection of fusion levels were noted according to the curve type and pattern. Even with similar deformity correction priorities, different surgeons may adopt quite different instrumentation configurations.

Due to the particular nature of spinal instrumentation, one could not realistically expect testing different surgical strategies on the same patient. Computer modelling and simulations of patient-specific instrumentations have thus become an important means in assisting surgeons to assess and evaluate various instrumentation scenarios and workout an optimal solution so as to maximize a given patient’s benefit. To do so, extensive research work has been conducted in computer biomechanical modelling and simulations of spinal instrumentations. However, patient-specific optimization technique which may be used in a clinical context is still absent.

For the above reason, the purpose of this study was to develop an optimization model to assist surgeons to determine the instrumentation configurations which are the most adaptive to achieve their particular correction objectives for their particular patient. Then, how instrumentation strategies vary with the correction objectives was examined.

## Methods

**Figure 1:**
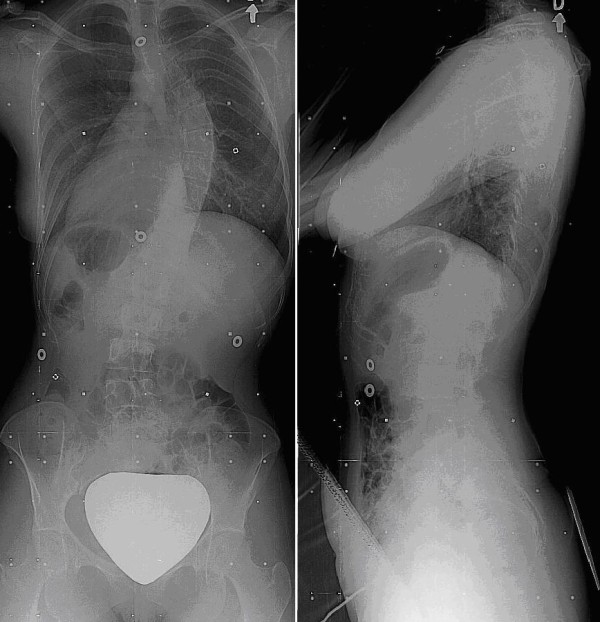
Preoperative posteroanterior and lateral radiographs of the patient.

A 16 year old female with AIS, candidate for surgical treatment was selected for analysis (Figure 1). This patient had a Lenke 2B curve type with a 51° left proximal thoracic curve, a 56° right main thoracic curve, a 38° left lumbar curve, thoracic kyphosis of 22°, and lumbar lordosis of 44°.

### Corrective objective function

The global spinal curve correction was quantified by an objective function Ф that was formulated using 12 different geometric measures describing the 3D spinal deformities and was arranged to minimize the number of instrumented levels (maximize the remaining mobility). The following coronal and sagittal measures were taken by following the Spinal Deformity Study Group (SDSG) Radiographic Measurement Manual [11]:

In the coronal plane:

• Proximal thoracic (PT) Cobb angle (θ_PT_)

• Main thoracic (MT) Cobb angle (θ_MT_)

• Thoracolumbar/lumbar (TL/L) Cobb angle (θ_TL/L_)

• Apical vertebra translation (X_AVT_)

In the sagittal plane:

• Thoracic kyphosis (θ_TK_)

• Lumbar lordosis (θ_LL_)

In addition, the following measures were used in the transverse plane:

• Apical vertebral rotation of the PT curve (θ_AVR-PT_)

• Apical vertebral rotation of the MT curve (θ_AVR-MT_)

• Apical vertebral rotation of the TL/L curve (θ_AVR-TL/L_)

• Orientation of the plane of maximum curvature of the PT curve (θ_PMC-PT_)

• Orientation of the plane of maximum curvature of the MT curve (θ_PMC-MT_)

• Orientation of the plane of maximum curvature of the TL/L curve (θ_PMC-TL/L_)

For the simulated instrumented spine, Cobb angles were calculated as the angles between the perpendicular lines to the spine curve at the inflexion points. The apical vertebral translation (AVT) was determined as the horizontal distance in centimeters measured between the midpoint of the apical vertebra (T8 in this study) and the C7 vertebra plumb line. The thoracic kyphosis was measured between the upper end plate of T4 and the lower end plate of T12. The lumbar lordosis was measured as the angle formed between the upper end plate of the T12 and the lower end plate of L5. The apical vertebral rotation was measured using the method based on the pedicle position by Stokes [12]. The orientation of the plane of maximum deformity for each spine segment was calculated as the angle between the planes defined by the respective apical and end vertebrae with the sagittal plane [13].

The objective function Ф was computed as the sum of the weighted square of the ratio of these descriptors over their initial values with the introduction of a mobility factor defined as the ratio of the number of unfused vertebrae (F) over the maximum number of unfused vertebrae in all the strategies (F^0^). The choice of the square of the ratio was from the consideration of making each descriptor positive and dimensionless, i.e. without an associated physical unit so that the weighted summation of descriptors of different natures can be performed to form the objective function of a minimization problem. In this way, before the spinal instrumentation, the ratios of all descriptors were equal to 1, allowing consistency for different cases and numerical robustness of the solution of the optimization. Each term in the objective function was multiplied by a weighting factor that was specified independently by eleven experienced spine surgeons who are fellows of the Scoliosis Research Society (SRS) and also members of the Spinal Deformity Study Group (SDSG), according to their importance for an optimal 3-D correction (Table 1). The objective function is thus as follows:

**Table 1:** Weights assigned by the eleven surgeons (S1-S11) to the terms of the objective function of correction

		**S1**	**S2**	**S3**	**S4**	**S5**	**S6**	**S7**	**S8**	**S9**	**S10**	**S11**
**Global weights (%)**	**Symbol**											
Correction in the Coronal plane	W1	30	50	30	45	30	20	60	30	25	50	30
Correction in the Sagittal plane	W2	30	20	30	45	30	50	30	30	10	20	10
Correction in the Transverse plane	W3	20	10	20	10	20	20	10	20	25	20	40
Mobility (Nb of unfused/saved vertebrae)	W4	20	20	20	0	20	10	0	20	40	10	20
**Specific weights assigned to the Coronal Plane (%)**												
Proximal thoracic Cobb (PT)	a1	10	15	5	5	5	20	30	5	5	5	25
Main Thoracic Cobb (MT)	a2	50	40	35	30	45	20	30	60	45	45	25
Thoraco-lumbar/Lumbar Cobb (TL/L)	a3	0	15	35	35	20	20	30	5	25	5	25
Apical Vertebra Translation	a4	40	30	25	30	30	40	10	30	25	45	25
**Specific weights assigned to the Sagittal plane (%)**												
Thoracic Kyphosis	b1	60	50	50	50	50	80	50	40	50	100	30
Lumbar Lordosis	b2	40	50	50	50	50	20	50	60	50	0	70
**Specific weights assigned to the transverse plane (%)**												
Apical Vertebral Rotation (PT)	c1	10	10	5	5	5	20	17	0	10	5	10
Apical Vertebral Rotation (MT)	c2	30	30	25	25	40	40	17	30	30	40	35
Apical Vertebral Rotation (TL/L)	c3	5	10	25	25	40	10	16	10	10	5	15
Orientation – plane of max. curvature (PT)	c4	25	10	15	15	5	10	17	0	10	5	10
Orientation – plane of max. curvature (MT)	c5	25	30	15	15	5	10	17	30	30	40	15
Orientation – plane of max. curvature (TL/L)	c6	5	10	15	15	5	10	16	30	10	5	15

(1) $$\begin{array}{l} \phi =W_{1}\cdot [a_{1}\cdot {\left(\frac{\theta_{\mathrm{PT}}}{\theta_{\mathrm{PT}}^{0}}\right)}^{2}+a_{2}\cdot {\left(\frac{\theta_{\mathrm{MT}}}{\theta_{\mathrm{MT}}^{0}}\right)}^{2}+a_{3}\cdot {\left(\frac{\theta_{TL/L}}{\theta_{\mathrm{LL}}^{0}}\right)}^{2}+a_{4}\cdot {\left(\frac{X_{\mathrm{AVT}}}{X_{\mathrm{AVT}}^{0}}\right)}^{2}]\\ \;+W_{2}\cdot [b_{1}\cdot {\left(\frac{\theta_{\mathrm{TK}}-\theta_{\mathrm{TK}}^{n}}{\theta_{\mathrm{TK}}^{0}-\theta_{\mathrm{TK}}^{n}}\right)}^{2}+b_{2}\cdot {\left(\frac{\theta_{\mathrm{LL}}-\theta_{\mathrm{LL}}^{n}}{\theta_{\mathrm{LL}}^{0}-\theta_{\mathrm{LL}}^{n}}\right)}^{2}]\\ \;+W_{3}\cdot [c_{1}\cdot {\left(\frac{\theta_{PMC-PT}}{\theta_{PMC-PT}^{0}}\right)}^{2}+c_{2}\cdot {\left(\frac{\theta_{PMC-MT}}{\theta_{PMC-MT}^{0}}\right)}^{2}+c_{3}\cdot {\left(\frac{\theta_{PMC-TL/L}}{\theta_{PMC-TL/L}^{0}}\right)}^{2}+c_{4}\cdot {\left(\frac{\theta_{AVR-PT}}{\theta_{AVR-PT}^{0}}\right)}^{2}+c_{5}\cdot {\left(\frac{\theta_{AVR-MT}}{\theta_{AVR-MT}^{0}}\right)}^{2}+c_{6}\cdot {\left(\frac{\theta_{AVR-TL/L}}{\theta_{AVR-TL/L}^{0}}\right)}^{2}]\\ \;+W_{4}\cdot [{\left(\frac{F^{0}}{F}\right)}^{2}] \end{array}$$

where W_1_-W_3_ are the weights assigned for the correction of descriptors in the coronal, sagittal and transverse planes respectively, W_4_ is that assigned for mobility, and a_1_-a_4_, b_1_-b_2_, and c_1_-c_6_ are assigned to individual parameters in 3 different planes. The angle θ^0^ was defined as the preoperative angle. The ‘normal’ thoracic kyphosis (θ^n^_TK_) and lumbar lordosis (θ^n^_LL._) were defined as arbitrary values within the normal ranges with their absolute differences from the patient’s preop values greater than 5° to avoid numerical instability arising from small denominators [14,15]. From the same numerical consideration, initial values which were less than 5° were rounded to 5°.

### Simulation model and optimization technique

In order to search for the most favorable instrumentation configurations for the correction objectives given by a surgeon, we used an optimization approach to minimize the objective function. Details of the optimization approach have been presented in [16], and are here summarized. This optimization method used six instrumentation design variables: the upper instrumented vertebra (UIV), the lower instrumented vertebra (LIV), the number, type and location of implants and the rod shape. These instrumentation parameters were manipulated in a uniform experimental design (U-type) [17,18] framework which was linked to a patient-specific biomechanical model implemented in a spine surgery simulator (S3) [16,19-21].

The simulator S3 allowed computing and analyzing the effects of an instrumentation strategy for a particular patient. First of all, the coronal and lateral numerical radiographs of the patient wearing a small calibration plate were preoperatively acquired [22,23]. The two high resolution numerical images allowed the creation of the patient’s 3-dimensional (3D) spine geometry using a 3D multi-view reconstruction technique [22]. This was done by first identifying anatomical landmarks on each vertebra (e.g. the middle and corner points of vertebral endplates, the extremities of pedicles, transverse and spinous processes). Using an optimization procedure, these landmarks’ 3D coordinates were computed and then used as control points to register a detailed vertebral geometry through a free form deformation technique [22,24]. The accuracy for the pedicles and vertebral bodies are, on average, 1.6 mm (SD 1.1 mm) and 1.2 mm (SD 0.8 mm), respectively [24]. For a given scoliotic spine, the reconstruction variations for the computed geometric indices do not exceed 0.8° for Cobb angles, 5.3° for sagittal curves, and are 4-8° for vertebral axial rotation angle, all of which are within the error levels reported for equivalent 2-dimensional measurements used by clinicians [23-25]. Then a biomechanical simulation model was created using the reconstructed spinal geometry of the patient. Basically, the biomechanical model contains the vertebrae (from T1 to pelvis) connected by intervertebral structures that were modelled using flexible elements. The mechanical properties of these flexible elements were defined using experiment data and further adjusted to account for the patient specific spinal stiffness [26]. The implants (screws, hooks) were modelled as rigid bodies while the implant-vertebra links were modeled as generalized non linear stiffness elements that restrained mobility in rotation and in translation. The stiffness coefficients were approximated using in-house experimental data on instrumented cadaveric vertebrae, but its parametric formulation will allow the use of more detailed data when available in the future. Boundary conditions were applied to represent the state of the patient spine on the surgical table. All degrees of freedom, except sagittal plane rotation, were fixed at the pelvis. At T1, the vertebra was allowed to translate and rotate freely in the frontal plane.

In this study, the involved corrective manoeuvres were the rod attachment, rod derotation, and compression/distraction. To simulate the rod attachment manoeuvre, forces and torques were gradually applied between the rod and the targeted implant to translate and pivot the rod until it is fully engaged into the half cylindrical surface of the implant (tulip top design of the implant head). Cylindrical joints were then created to connect the implant head to the rod. For the rod derotation manoeuvre, a torque was gradually applied on the rod up until its profile was parallel to the sagittal plane. As the rod was derotated, the implants were free to slide along and rotate about the rod central axis. The compression/distraction manoeuvre was simulated by gradually applying a force between the two identified implants up until a specified distance was achieved.

In terms of coronal and sagittal plane Cobb angles and apical vertebral axial rotation angles, model validation has been performed in our previous works. This was done by simulating the documented spinal instrumentations of ten AIS patients and comparing the Cobb and rotation angles computed on the reconstructed postoperative spine models and those on the resulting spine geometries of the simulations. For the instrumented spinal segments, the differences did not exceed 5°.

For each surgeon, 702 surgical configurations were generated to form the searching space. Using each configuration, instrumentation simulation was performed using S3 and for each configuration 12 geometric parameters were measured. Eleven equations were built from the linear regression coefficients. These equations were obtained and used to make a simplified model representing the 12 geometric measurements as a function of the six instrumentation variables. These equations were entered into the objective function Ф(x). Once the approximation model describing the relationship between design variables and the objective function was obtained, the minimum was found using the Matlab Optimization Toolbox (MathWorks, USA). To solve the optimization problems, the function “fmincon” [27] was used.

Using this optimization approach, the most favorable strategy for the correction objectives of each surgeon was obtained, thus the influence of the eleven different correction objectives on the optimal surgical strategy was evaluated. Statistical analyses were conducted using Statistica software (StatSoft, Inc. 2001. data analysis software system). Difference in the number of fusion levels used between the instrumentation configurations of the surgeons was evaluated with an analysis of variance (ANOVA) one-way. The effect of correction objectives on instrumentation choices (the number of instrumented levels, upper and lowest fusion levels, the number, type and location of implants) was assessed with ANOVA one-factor repeated measures. Statistical significance was set at P<0.05.

## Results

**Figure 2:**
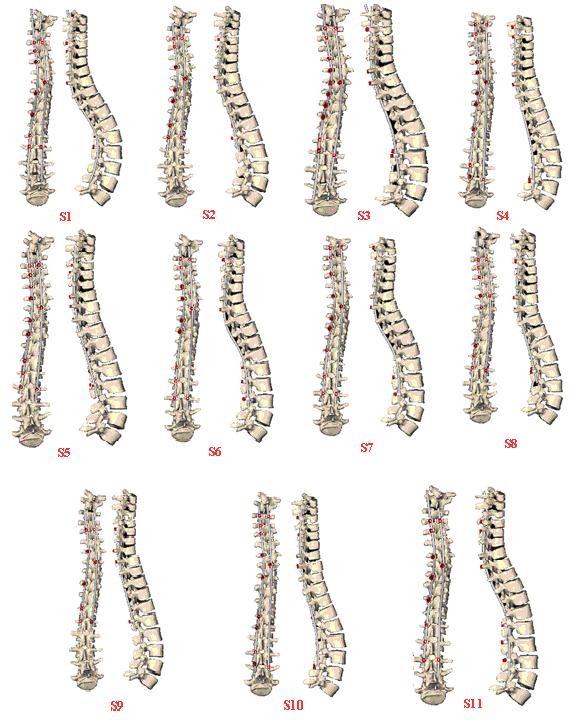
Resulting correction and optimal instrumentation configuration obtained from the simulation of the eleven optimized configurations for the same patient.

The resulting instrumentation configurations obtained from the optimization procedure are summarized in Table 2 and Figure 2.

**Table 2:** Resulting instrumentation parameters from the optimization simulations for the eleven optimal strategies based on the correction objectives provided by the eleven surgeons (S1-S11)

		**S1**	**S2**	**S3**	**S4**	**S5**	**S6**	**S7**	**S8**	**S9**	**S10**	**S11**
Type of Implants		Screw	Screw	Screw	Screw	Screw	Screw	Screw	Screw	Screw	Screw	Screw
Number of Implants		10	10	13	13	12	12	12	11	8	12	11
Number of fused levels		10	9	11	15	10	12	14	10	8	14	11
Upper instrumented vertebra (UIV)		T4	T4	T3	T2	T4	T3	T2	T4	T5	T3	T4
Lowest instrumented vertebra (LIV)		L2	L1	L2	L4	L2	L3	L3	L2	L1	L4	L3
Shape of the rod	Thoracic curve	30°	20°	20°	20°	20°	20°	30°	20°	20°	30°	30°
	Lumbar curve	30°	45°	30°	45°	30°	30°	30°	45°	45°	45°	45°

Overall, the correction objectives (Table 1) have a significant influence (p< 0.05) on the resulting instrumentation configurations (Table 2). For example, the correction objectives for the number of instrumented levels (mobility) that were different between the eleven surgeons (range from 0% to 40%; Table 1), along with the other correction objectives, resulted in statistically different (p < 0.001) numbers of instrumented levels (from 8 to 15; mean: 11.2; STD 2.1) (Table 2). All other instrumentation objectives were also statistically different (p < 0.001). The upper instrumented level ranged from T2 to T5, while the lowest instrumented vertebra ranged from L1 to L4 (Table 2 and Figure 2). The optimal number of screws ranged from 8 to 13 (mean: 11.2; STD 1.4). The resulting shape of the rod and the position of the screws were also different between the eleven simulated optimized strategies (Figure 2).

There are significant (p<0.001) differences in the simulated curve correction for the eleven instrumentation configurations. The resulting Cobb angle varied between 26° and 40° for the PT region, between 12° and 24° for the MT segment, and between 13° and 27° for the TL/L segment. A difference was also noted in the resulting simulated correction in the sagittal and transverse planes. The computed postoperative Cobb angles varied from 20° to 29° for the kyphosis, from 30° to 42° for the lordosis, and from 38° to 51° for the orientation of the plane of maximum deformity with respect to the sagittal plane (Table 3).

**Table 3:** Resulting correction obtained from the simulation of the eleven optimized configurations for the same patient

	**Preoperative**	**S1**	**S2**	**S3**	**S4**	**S5**	**S6**	**S7**	**S8**	**S9**	**S10**	**S11**
Proximal thoracic Cobb	51º	40°	36°	40°	28°	33°	34°	29°	39°	36°	37°	26°
Main thoracic Cobb	56º	18°	19°	24°	17°	16°	17°	20°	12°	19°	15°	17°
Thoraco-lumbar/Lumbar Cobb	38º	24°	25°	27°	13°	24°	23°	23°	24°	25°	18°	23°
Thoracic Kyphosis	22°	27°	29°	20°	20°	21°	26°	28°	23°	22°	21°	20°
Lumbar Lordosis	44°	37°	40°	32°	37°	35°	33°	34°	30°	42°	35°	34°
Orientation – plane of max. curvature	58°	47°	46°	38°	45°	43°	49°	40°	40°	51°	42°	40°

## Discussion

The study proposed an optimization model to assist surgeons to search for the most effective instrumentation configurations according to their particular correction objectives. Evaluation was performed on the effect of different surgeon-specified correction objectives on the correction of the spinal curves for the same AIS patient, using a patient-specific biomechanical model implemented in a spine surgery simulator. It was shown that different correction objectives lead to different instrumentation strategies (e.g. fusion levels, implant positioning and rod shape), and obviously different correction results. The findings are similar to those reported in [21] wherein it was shown that different instrumentation strategies produced rather different surgical results. Our study further highlights another source of variability in the surgical correction process, i.e. the correction objectives based on which the preoperative planning of surgery in AIS. This study demonstrates the degree of variability among clinicians with regard to what constitutes desirable correction objectives. Although an optimal instrumentation strategy can be identified for a particular set of correction objectives, perhaps the most challenging aspect is to ensure that the correction objectives are well tailored to each individual patient's particular deformity.

Variability in the selection of instrumentation strategies has already been reported in previous studies [1,9]. The findings of this study confirm these previous findings and further identify another element associated to the variability that can be attributed to the objectives of surgical correction. It also emphasizes the need for a standardized decision-making protocol (procedure) to minimize the inherent variability in defining the correction objectives of AIS patients.

Computer simulations constitute an assisted-decision making approach that is versatile, fast (< 1 hour for the 702 iteration process per surgeon) and feasible, and that can be easily adapted to surgeon-specific preferences. We demonstrated the possibility of using a simulator to optimize the instrumentation strategy for a specific patient and specific correction objectives, and to evaluate the effect of how a change in the correction objective influences the strategy and thus the surgical outcome.

Simplifications and approximations made in the development of the spinal instrumentation simulator put some limitations on this study. One of the limitations is the choice of the boundary conditions applied to the spine model (partially fixing T1 and pelvis), which represents a simplification of the real spine wherein the vertebral levels are not entirely fixed. Including the cervical vertebrae instead of T1 could improve accuracy and provide a more realistic behaviour of the non-instrumented spinal segment. However, this will not account for the balance control and postoperative decompensation. Balance-related parameters in the coronal and sagittal planes are essential goals of surgical correction [9] and further studies are required to elucidate their role.

The high heterogeneity of the deformities and mechanical properties of the scoliotic spines and the great variation of the instrumentation strategies among surgeons made the model validation extremely challenging. The model validation was still limited to the instrumented spinal segments with the prediction errors being within the accepted range of variations of radiographic measurements performed by clinicians. For the non-instrumented spinal segments, the confidence levels on the simulator’s predictions have yet to be established. Consequently, there are potential limitations when running simulations of a great number of instrumentation strategies involving various scenarios of non-instrumented spinal segments. This study was also limited by the fact that only the geometric aspects of the scoliosis instrumentation were considered and modelled into the objective function. Other biomechanical aspects, e.g. bone-screw force levels, risk factors of the occurrence and development of proximal junctional kyphosis, etc. are yet to be studied. In addition to the aforementioned limitations in the modelling, solution errors may also come from the evaluation of the objective function using the still more simplified model representing the 12 geometric measurements as a function of the six instrumentation variables.

Through the development of computer modeling, simulations and optimization techniques, as well as their application on a single AIS case, this study highlights the inherent variability factors associated with surgical-planning and decision-making in AIS instrumentation. The limitations on the generalization of the findings reside in the fact that the influences of the curve type, spine stiffness, and deformity magnitude, etc. have not yet been explored. Full study through statistically significant number of cases and deformity variation is yet to be conducted to make the simulator and optimization technique ready for use by clinicians.

## Conclusions

This study demonstrates that different surgeon-specified correction objectives produced different instrumentation strategies for the same patient. It still highlights the inherent variability factors associated with surgical-planning and decision-making in AIS. To our knowledge, this is the first study to analyze the effect of different correction objectives on the surgical outcome. The next step is to apply the simulation methods to a larger cohort of scoliotic patients and further exploit the potential of the simulator in facilitating the surgical decision-making.

## Abbreviations

AIS: Adolescent idiopathic scoliosis; SD: Standard deviation; 3D: Three-dimension; SDSG: Spinal Deformity Study Group; SRS: Scoliosis Research Society; PT: Proximal thoracic; MT: Main thoracic; TL/L: Thoracolumbar/lumbar; AVT: Apical vertebral translation; UIV: Upper instrumented vertebra; LIV: Lower instrumented vertebra; S3: Spine surgery simulator.

## Competing interests

The authors declare that there are no financial and non-financial competing interests related to the publication of this manuscript.

## Authors’ contributions

YM and CEA have made substantial contributions to the study design, computer modelling, numerical simulations, analysis and interpretation of data. XW, AS, and HL have significantly contributed in drafting the manuscript and revising it critically for important intellectual content. All authors read and approved the final manuscript.
